# Hypoxia Impairs Muscle Function and Reduces Myotube Size in Tissue Engineered Skeletal Muscle

**DOI:** 10.1002/jcb.25982

**Published:** 2017-05-15

**Authors:** Neil R.W. Martin, Kathyrn Aguilar‐Agon, George P. Robinson, Darren J. Player, Mark C. Turner, Stephen D. Myers, Mark P. Lewis

**Affiliations:** ^1^ School of Sport, Exercise and Health Sciences Loughborough University Loughborough UK; ^2^ Department of Sport and Exercise Sciences University of Chichester Chichester UK

**Keywords:** ATROPHY, mTORC1, UBIQUITIN‐PROTEASOME, OXYGEN

## Abstract

Contemporary tissue engineered skeletal muscle models display a high degree of physiological accuracy compared with native tissue, and therefore may be excellent platforms to understand how various pathologies affect skeletal muscle. Chronic obstructive pulmonary disease (COPD) is a lung disease which causes tissue hypoxia and is characterized by muscle fiber atrophy and impaired muscle function. In the present study we exposed engineered skeletal muscle to varying levels of oxygen (O_2_; 21–1%) for 24 h in order to see if a COPD like muscle phenotype could be recreated in vitro, and if so, at what degree of hypoxia this occurred. Maximal contractile force was attenuated in hypoxia compared to 21% O_2_; with culture at 5% and 1% O_2_ causing the most pronounced effects with 62% and 56% decrements in force, respectively. Furthermore at these levels of O_2_, myotubes within the engineered muscles displayed significant atrophy which was not seen at higher O_2_ levels. At the molecular level we observed increases in mRNA expression of MuRF‐1 only at 1% O_2_ whereas MAFbx expression was elevated at 10%, 5%, and 1% O_2_. In addition, p70S6 kinase phosphorylation (a downstream effector of mTORC1) was reduced when engineered muscle was cultured at 1% O_2_, with no significant changes seen above this O_2_ level. Overall, these data suggest that engineered muscle exposed to O_2_ levels of ≤5% adapts in a manner similar to that seen in COPD patients, and thus may provide a novel model for further understanding muscle wasting associated with tissue hypoxia. J. Cell. Biochem. 118: 2599–2605, 2017. © 2017 The Authors. *Journal of Cellular Biochemistry* Published by Wiley Periodicals, Inc.

Tissue engineered skeletal muscle shows excellent structural and functional similarities to in vivo tissue [Cheng et al., [Link]], with a variety of published models capable of generating aligned myotubes surrounded by biologically relevant matrices, and able to produce contractile force. As such, engineered skeletal muscle models are proving useful tools for physiological investigations concerned with muscle adaptation [Khodabukus and Baar, 2015; Khodabukus et al., 2015], and may also have utility in furthering our understanding of diseases characterized by alterations in muscle phenotype. However, at present there is little data concerning the manipulation of engineered muscle to replicate diseased tissue.

Chronic obstructive pulmonary disease (COPD) is a lung disease which leads to a reduction in tissue oxygenation and therefore a state of tissue hypoxia. Skeletal muscle of COPD patients is characterized by reduced fiber size compared to healthy individuals (muscle atrophy), and a reduction in muscle strength [Mador and Bozkanat, 2001], which ultimately increases morbidity and mortality in these individuals [Swallow et al., 2007]. The loss of muscle size is likely due to alterations in molecular pathways which regulate muscle growth and breakdown. The mTORC1 signaling pathway is a well characterized anabolic pathway which is activated in skeletal muscle by nutrients, growth factors, and muscle loading [Kimball, 2014], resulting in increased mRNA translational capacity and therefore muscle growth. Conversely, the ubiquitin proteasome system is a catabolic pathway, which is heavily implicated in the loss of skeletal muscle size in scenarios such as disuse and disease [Bodine et al., 2001; Lecker et al., 2004], and the two E3 ubiquitin ligases Muscle Ring Finger 1 (MuRF‐1) and Muscle Atrophy F‐box (MAFbx) have been shown to induce muscle atrophy when overexpressed or prevent atrophy when knocked out using animal and cell culture models [Bodine et al., 2001]. Skeletal muscle of COPD patients has been reported to express elevated levels of MuRF‐1 and MAFbx, and reductions in mTORC1 signaling proteins such as p70S6 kinase and 4EBP‐1 [Favier et al., 2010], and thus both reductions in protein synthesis and increases in protein breakdown likely drive the loss of muscle size in this disease state.

While individuals suffering from COPD display severe tissue hypoxia, they also exhibit systemic inflammation [Takabatake et al., 2000], negative energy balance [Vermeeren et al., 1997], and oxidative stress [Rahman et al., 1996], which are all confounding factors which may contribute to the muscle atrophy observed in this population. However, there is a growing body of literature to suggest that hypoxia itself acts as a stimulus for muscle atrophy. For example, de Theije et al. exposed rats to step wise reductions in oxygen (O_2_) levels and found significant reductions in muscle size which were greater than those observed in a pair fed group designed to account for the reductions in appetite associated with hypoxia. This loss of muscle mass was also associated acutely with reduced p70S6 kinase phosphorylation, and chronically with sustained elevations in MuRF‐1 and MAFbx mRNA levels [de Theije et al., 2013]. Similarly, in vitro studies have observed loss of cell size when myotubes are exposed to low O_2_ conditions [Caron et al., 2009], and impaired muscle regeneration when cells differentiate in hypoxia [Di Carlo et al., 2004; Yun et al., 2005]; an effect also mirrored in vivo [Chaillou et al., 2014].

Human skeletal muscle normally experiences O_2_ tension of approximately 35 mmHg [Richardson et al., 2006] or 5% O_2_, which is considered physiological tissue hypoxia, and thus in pathological hypoxia such as that associated with COPD O_2_ levels are further reduced in comparison. In vitro however, cells are routinely cultured at 21% O_2_, and therefore in comparison any reductions in O_2_ levels below this may be sensed by the cells as a hypoxic stimulus. The majority of skeletal muscle in vitro investigations to date have delivered hypoxia in the region of 1–5% O_2_ [Chakravarthy et al., 2001; Di Carlo et al., 2004; Yun et al., 2005; Caron et al., 2009; Martin et al., 2009; Li et al., 2011; Liu et al., 2012], thus spanning the physiological/pathological hypoxia spectrum. However, the effect of varying O_2_ concentrations on skeletal muscle size and function, and the influence on catabolic/anabolic signaling pathways has not yet been explored.

In the present investigation, we therefore, cultured tissue engineered skeletal muscle acutely (24 h) under varying levels of hypoxia, and sought to determine whether; (i) engineered muscle responds to reductions in %O_2_ in a similar manner to skeletal muscle (i.e., atrophy) and therefore may provide a useful model of hypoxia associated skeletal muscle disease, and (ii) at what %O_2_ the loss of muscle size and function is apparent. We hypothesized that a step wise reduction in muscle force and atrophy would be observed as the %O_2_ was reduced below 21% since cells in culture would view this as a hypoxic stimulus. We also predicted that these phenotypic observations would be underpinned by increases in markers of ubiquitin proteasome activity and a reduction in mTORC1 signaling.

## METHODOLOGY

### CELL CULTURE

C2C12 murine myoblasts (ECACC, Sigma–Aldrich, UK) were used for all experimentation, and cultured in T80 flasks (Nunc™, Fisher Scientific, UK) maintained in a humidified 5% CO_2_ incubator (HERAcell 240i, Thermo Fisher, UK). Cells were maintained in growth media (GM) consisting of high glucose DMEM (Fisher Scientific, UK) supplemented with 20% FBS (Dutscher Scientific, UK), 100 U/ml penicillin, and 100 μg/ml streptomycin (Fisher Scientific) until 80% confluent, at which point cells were enzymatically detached from the flasks with Trypsin‐EDTA and counted using the trypan blue exclusion method. Thereafter cells were either re‐plated for further subculture, or used for experimentation. All experiments were conducted using cells which had undergone fewer than 10 passages since receipt.

### TISSUE ENGINEERING SKELETAL MUSCLE AND EXPERIMENTAL DESIGN

**Figure 1:**
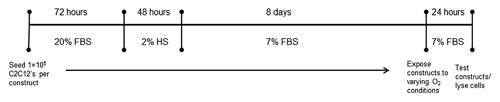
Experimental time line used to explore the atrophic effects of 24 h of varying oxygen levels in tissue engineered skeletal muscle. Engineered muscle was cultured for 13 days prior to exposure to 21%, 15%, 10%, 5%, or 1% oxygen for a further 24 h at which point muscles were analyzed appropriately.

Engineered muscle constructs were fabricated as previously described [Martin et al., 2015]. Briefly, two 6 mm sutures were pinned into PDMS (Sylgard 184 Elastomer, Dow Corning, UK) coated 35 mm plates 12 mm apart using 0.15 mm minutien pins (Entomoravia, Czech Republic). Plates were sterilized using ultraviolet light and washing with 70% ethanol and subsequently left to dry for 3 h. Each plate then received 500 μl of GM containing 10 U/ml thrombin (Sigma–Aldrich) and 80 μg/ml aprotinin (Sigma–Aldrich) which was spread evenly over the surface of the plate ensuring that the sutures were fully covered. 200 μl of 20 mg/ml stock fibrinogen (Sigma–Aldrich) solution was then added to the plate, and was agitated gently to ensure even distribution and then left to incubate for 10 min at room temperature before being transferred to the incubator (37°C) for 1 h. Thereafter, 1 × 10^5^ C2C12 myoblasts were seeded on to the surface of the construct in GM supplemented with 0.25 mg/ml 6‐aminocaproic acid (Sigma–Aldrich) which was replaced daily for the first 4 days of the culture period. On day 4, GM was replaced with Differentiation media (DM) consisting of high glucose DMEM supplemented with 2% horse serum (Sigma–Aldrich), 100 U/ml penicillin, and 100 μg/ml streptomycin and 0.5 mg/ml 6‐aminocaproic acid. Following 2 days in DM, the media was changed to maintenance media (MM) containing 7% FBS (Dutscher Scientific) in order to help prevent excessive fibrin degradation [Khodabukus and Baar, 2009], which was replaced daily for the remainder of the culture period. At day 13, following media replenishment constructs were exposed to either relative hypoxia (1%, 5%, 10%, or 15% O_2_) or normoxia (21% O_2_) for the final 24 h of the culture period (see Fig. 1). To achieve this, normoxic constructs were returned back to the standard incubator while hypoxic constructs were transferred to a separate incubator where the O_2_ levels can be altered through the addition of nitrogen gas (MCO‐5M, Sanyo, UK).

### ASSESSMENT OF MUSCLE FUNCTION

To determine if hypoxia could impact upon maximal skeletal muscle force production, after 24 h of exposure constructs were removed from the respective condition, washed once in a krebs ringer HEPES buffer (KRH; 10 mM HEPES, 138 mM NaCl, 4.7 mM KCl, 1.25 mM CaCl_2,_ 1.25 mM MgSO_4_, 5 mM Glucose, 0.05% bovine serum albumin in dH_2_0) and attached to a model 403A Aurora force transducer (Aurora Scientific, UK). Following the addition of 4 ml of KRH buffer, stainless steel electrodes were positioned either side of the engineered muscle in order to allow for electric field stimulation. Maximal tetanic force was generated using labVIEW 2012 software (National Instruments, UK) programmed to deliver a 1 s pulse train at 100 Hz and 3.6 v/mm. Data was acquired using a PowerLab 8/35 (ADInstruments, UK) at a sampling rate of 1 KHz, and was analyzed with associated LabChart 8 software.

### HISTOCHEMISTRY

In order to assess cellular morphology, immediately after functional testing engineered muscles from normoxic and hypoxic conditions were fixed by the drop wise addition of ice‐cold methanol‐acetone solution. Subsequently constructs were removed from their sutures and adhered to poly‐L‐lysine coated microscope slides and ringed with PAP pen (Sigma–Aldrich) before being permeablized with 1× Tris buffered saline (TBS: 0.5M) and 0.2% Triton x‐100 (Fisher Scientific, UK) for 2 h. Constructs were then incubated in rhodamine conjugated phalloidin (Fisher Scientific) diluted 1:200 in TBS to label F‐actin, and DAPI (Sigma–Aldrich) diluted 1:1000 in order to label cellular nuclei, and were incubated at room temperature in the dark for 3 h. Following 4 washes in distilled water, constructs were mounted on to glass coverslips using a drop of Fluoromount™ medium (Sigma–Aldrich). Images were captured using a Leica DM2500 Fluorescent microscope at 40 × magnifications and analysis was conducted using Image J software (NIH, USA), with a minimum of five images and 40 myotubes analyzed per engineered muscle.

### RNA EXTRACTION AND RT‐qPCR

After 24 h of exposure to varying oxygen conditions, constructs were homogenized in 500 μl of TRI Reagent® (Sigma–Aldrich) using an electronic stick homogenizer (IKA T10, Fisher Scientific), and RNA was extracted thereafter according to the manufacturer's instructions. RNA concentration and quality was assessed by UV spectroscopy at optical densities of 260 and 280 nm using a Nanodrop 2000 spectrophotometer (Thermo Fisher). Only RNA with a 260/280 ratio of between 1.8 and 2 was used for RT‐qPCR analysis.

mRNA expression of MuRF‐1 and MAFbx was determined using One‐step RT‐qPCR. Reactions were prepared in transparent 384 well plates (Applied Biosystems, Thermo Fisher Scientific, UK), consisting of 20 ng of RNA in 5 μl of nuclease free water, 0.1μl of Quantifast reverse transcription kit (Qiagen, UK), 0.1 μl of both forward and reverse primers (see Table I, Primers were obtained from Sigma–Aldrich, UK), and 4.7μl of SYBR Green mix (Qiagen) in order to create 10 μl final reaction volumes. Plates were then transferred to a Applied Biosystems Viia 7™ thermal cycler (Applied Biosystems) which was programmed to perform the following steps: 10 min hold at 50°C (reverse transcription), followed by a 5 min hold at 95°C (activation of “hot start” Taq polymerase), and cycling between 95°C for 10 s (denaturation) and 60°C for 30 s (annealing and extension). Fluorescence was detected after every cycle and data was analyzed using the 2^(−ΔΔCT)^ method making samples relative to one replicate 21% O_2_ control sample in each experiment and normalized to POLR2B levels.

**Table I:** Primer Sequences Used in This Study for the Detection of E3 Ubiquitin Ligase mRNA

Target mRNA	Primer sequence 5′‐3′	Product length	NCBI reference sequence
MAFbx	F = GTCGCAGCCAAGAAGAGAA	134	NM_026346.3
	R = CGAGAAGTCCAGTCTGTTGAA		
MuRF‐1	F = CCAAGGAGAATAGCCACCAG	67	NM_001039048.2
	R = CGCTCTTCTTCTCGTCCAG		
POLR2B	F = GAGAAAGGCTTGGTCAGA	148	NM_153798.2
	R = AATATCTTGGCGGCTCTT		

### IMMUNOBLOTTING

Following 24 h of exposure to varying O_2_ conditions, MM was removed from the engineered muscle, which was washed twice in PBS, removed from its sutures, blotted dry and frozen in liquid nitrogen, and stored at −80°C until further analysis. Samples were subsequently homogenized in RIPA lysis buffer (Fisher Scientific) containing a protease and phosphatase inhibitor cocktail (Fisher Scientific) and rotated for 1 h at 4°C before being centrifuged at 12,000 × *g* in order to remove insoluble material. The supernatant was transferred to a fresh tube and protein concentrations were determined using the Pierce 660 protein assay (Fisher Scientific). Protein was mixed with a 3 X laemmli buffer and boiled at 95°C for 5 min and equal volumes (10 μg) were loaded in to 8% SDS‐polyacrylamide gels and separated at 120 V. Proteins were transferred on to nitrocellulose membranes for 2 h at 0.35 A (GE healthcare, Fisher Scientific) and blocked in 5% BSA at 4°C for 90 min. Thereafter, membranes were washed three times in tris‐buffered saline + 0.1% tween (TBST) and incubated with phospho p70S6 kinase^Thr389^ (Cell Signalling Technologies, #9234, 1:1000) or total p70S6 kinase (Cell Signalling Technologies, #2708, 1:2000) overnight at 4°C. Following three further washes in TBST, membranes were incubated for 1 h at room temperature in HRP‐conjugated anti‐rabbit IgG secondary antibody (Sigma–Aldrich) diluted 1:1500 in TBST containing 5% skimmed milk powder before detection with chemilluminescence. Imaging and band quantification were conducted on a ChemiDoc imaging system (Bio‐rad, UK) using Quantity One image software (Version 4.6.8, Bio‐rad). Phosphorylation levels are normalized to total p70S6 kinase and α‐tubulin (Cell Signalling Technologies, #2125, 1:2000) abundance, and are presented as a fold change compared to a single control (21% O_2_) sample in each experiment.

### STATISTICAL ANALYSIS

All data are presented as mean ± SEM. Normality of distribution and homogeneity of variance in all data sets was determined using a Shapiro–Wilk test and Levene's tests, respectively. Data were subsequently analyzed using either One–Way ANOVA with LSD post‐hoc tests or Kruskall–Wallis tests and Mann–Whitney tests where data were not normally distributed. All analysis was conducted using SPSS version 22.

## RESULTS

### PHYSIOLOGICAL HYPOXIA IMPAIRS MAXIMAL CONTRACTILE FORCE PRODUCTION

**Figure 2:**
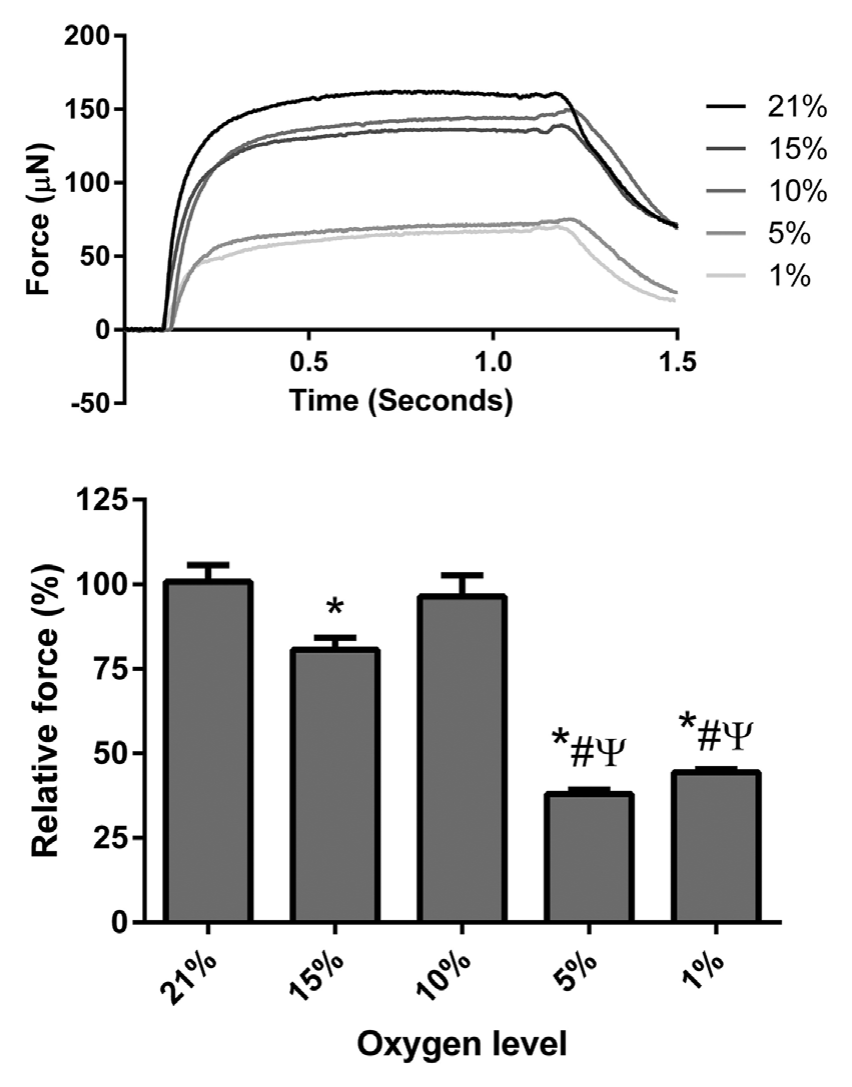
Maximal contractile force from engineered muscles cultured for 24 h at various levels of hypoxia. All hypoxic cultures were compared to 21% O_2_ control cultures within individual experiments to calculate relative force. Data are expressed as mean ± SEM for n = 3/4 engineered muscles. * indicates statistically different to 21% O_2_ (*P* ≤ 0.05), # indicates significantly different to 15% O_2_ (*P* ≤ 0.05), Ψ indicates statistically different to 10% (*P* ≤ 0.05).

Decreasing levels of O_2_ significantly affected maximal force output in engineered skeletal muscles (main effect for condition, *P* < 0.05) following 24 h of treatment at the end of the culture period. In the most extreme levels of physiological hypoxia tested, maximal force production was reduced by 56% and 62% in 1% and 5% O_2_ conditions, respectively (*P* < 0.05), compared to ambient (21%) control muscles (Fig. 2). By contrast, less extreme levels of hypoxia had a smaller impact on maximal force output, with 10% O_2_ leading to a 4% force decrement compared to ambient control muscles (*P* = 0.626), and 15% O_2_ associated with a 20% reduction in maximal force (*P *< 0.05, Fig. 2).

### REDUCED OXYGEN LEVELS ARE ASSOCIATED WITH MYOTUBE ATROPHY IN ENGINEERED SKELETAL MUSCLE

**Figure 3:**
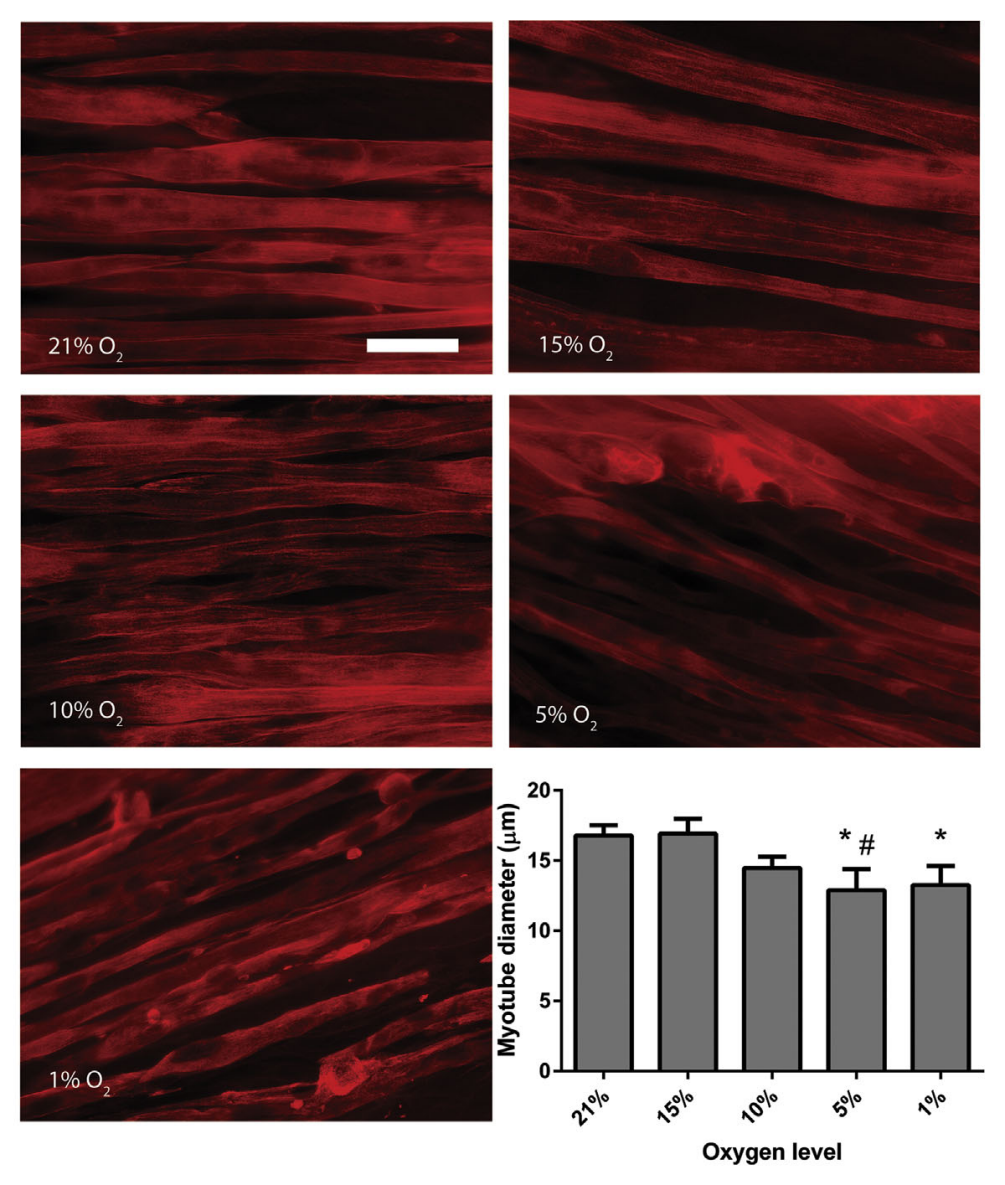
Myotube diameter in engineered muscles following 24 h of culture at various levels of hypoxia. Data are expressed as mean ± SEM for n = 4 engineered muscles. Scale bar = 20 μm. * indicates statistically different to 21% O_2_ (*P* ≤ 0.05), # indicates statistically different to 15% O_2_ (*P* ≤ 0.05).

Loss of myotube size (diameter) appeared to occur in a dose‐dependent fashion, with more extreme levels of hypoxia associated with significant atrophy (*P* < 0.05, Fig. 3). Culturing engineered muscles at 15% O_2_ had no effect on myotube diameter compared to 21% O_2_ control muscles (16.92 ± 1.06 vs. 16.80 ± 1.47 µm, *P* = 0.938). There was a small reduction in myotube diameter when engineered muscles were cultured at 10% O_2_ (14.47 ± 0.80 µm), which although did not reach statistical significance (*P* = 0.14) does represent a 14% reduction in myotube size compared to control muscles. However, when engineered muscles were cultured for 24 h in either 5% (12.90 ± 1.50 μm) or 1% (13.26 ± 1.36 μm) O_2_, there was a statistically significant reduction in myotube diameter (*P *< 0.05) which represented 23% and 21% reductions in myotube size, respectively.

### PHYSIOLOGICAL HYPOXIA INCREASES mRNA LEVELS OF MuRF‐1 AND MAFbx AND DAMPENS PHOSPHORYLATION OF p70S6 KINASE

**Figure 4:**
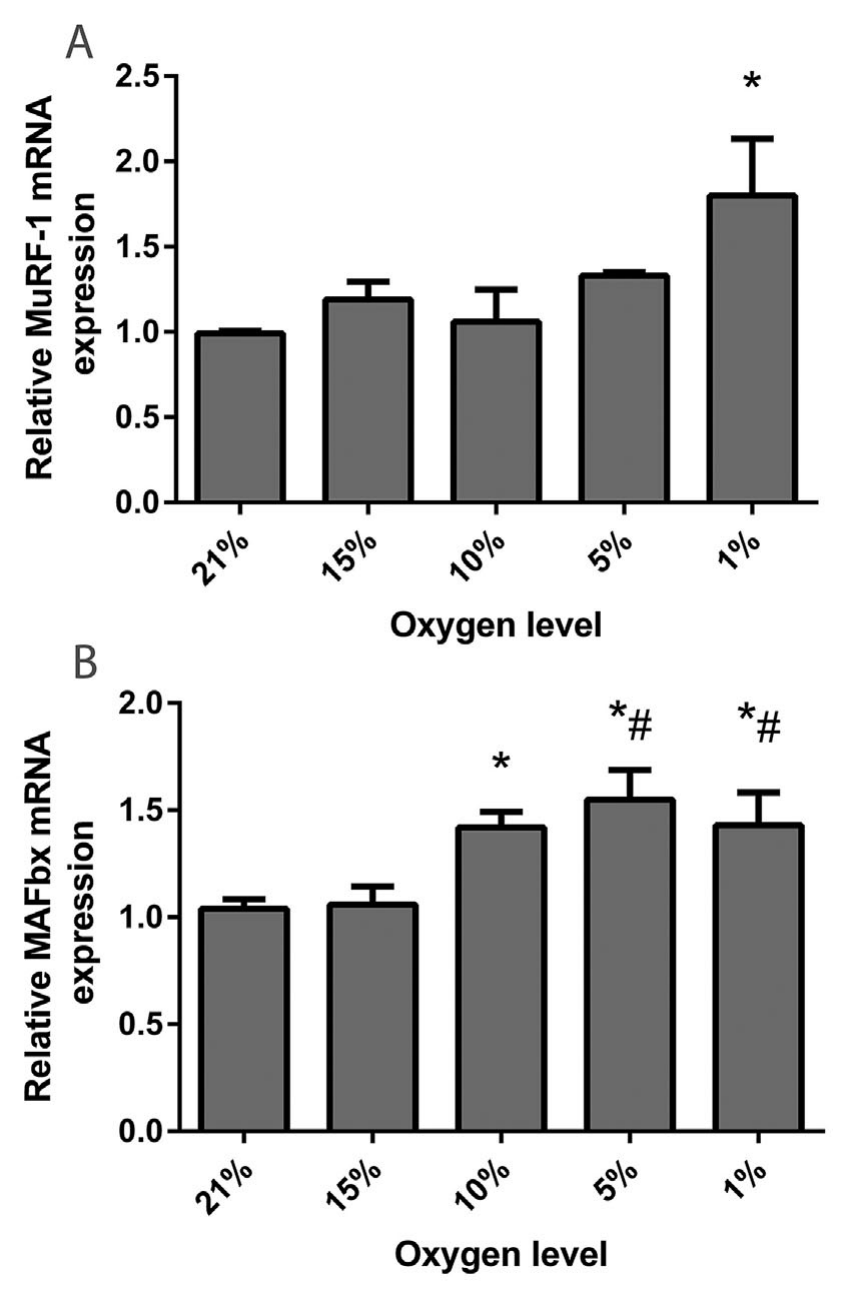
mRNA expression of E3 ubiquitin ligases following 24 h of culture at various levels of hypoxia. (A) Muscle Ring Finger‐1, and (B) Muscle Atrophy F‐box. Values are normalized to levels of POLR2B and made relative to 21% O_2_. Data are expressed as mean ± SEM from n = 4 engineered muscles. * indicates statistically different to 21% O_2_ (*P* ≤ 0.05) # indicates statistically different to 15% O_2_ (*P* ≤ 0.05).

**Figure 5:**
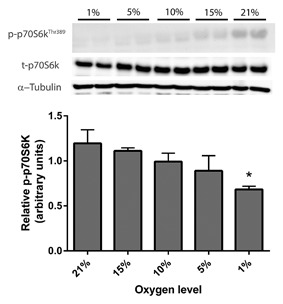
Phosphorylation of p70S6 kinase following 24 h of culture at various levels of hypoxia. Phosphorylated p70S6 kinase is normalized to the levels of total p70S6 kinase and α‐tubulin. Data are expressed as mean ± SEM for n = 4 engineered muscles. * indicates statistically different to 21% O_2_ (*P* ≤ 0.05). Note that the direction of the western blot is opposite to that of the representative graph.

mRNA levels of the E3 ubiquitin ligases, commonly used as markers of muscle atrophy/wasting, were elevated by physiological levels of hypoxia. MuRF‐1 mRNA (Fig. 4A) expression was significantly affected by physiological hypoxia (*P* < 0.05), with engineered muscle cultured in 5% (1.33 ±0.02) and 1% (1.80 ± 0.33) O_2_ showing elevated levels compared to controls in ambient O_2_ (0.99 ± 0.02, *P* < 0.05). MAFbx expression (Fig. 4B) was also significantly increased by hypoxia in engineered muscle (*P* < 0.05), and appeared to be more sensitive to low O_2_ levels since its levels were elevated by 10% (1.42 ± 0.07), 5% (1.55 ± 0.14), and 1% (1.43 ± 0.15) O_2_ compared with 21% control muscles (1.04 ± 0.04). Phosphorylation of p70S6k, a downstream kinase from mTOR was also altered by hypoxia (Fig. 5). While there was a step‐wise reduction in p70S6 kinase phosphorylation with lower O_2_ levels, only culture at 1% O_2_ significantly dampened this response (*P *< 0.05). No significant change in total levels of p70S6 kinase were observed (*P* = 0.238) although a slight reduction was associated with more extreme hypoxia (effect size r = 0.42).

## DISCUSSION

Engineered muscle models closely replicate the physiology and function of native tissue, however to date there is little data regarding their ability to replicate muscle from diseases associated with alterations in muscle phenotype. In this study we aimed to determine if exposure of engineered muscle to hypoxia in vitro could recreate tissue with similar characteristics to the atrophic muscle observed in diseases where tissue hypoxia is prevalent in vivo (e.g., COPD). We found that severe loss of muscle force and size were only associated with the lowest levels of O_2_ tested, which corresponded with changes in molecular markers of skeletal muscle loss.

Patients with COPD suffer from a loss of muscle strength of up to 50% compared with age matched healthy individuals [Mador and Bozkanat, 2001], and during acute exacerbations of COPD symptoms, muscle strength is known to further decrease [Gayan‐Ramirez and Decramer, 2013]. While there are likely a number of factors involved in the worsening of muscle performance (e.g., physical inactivity, inflammation, use of glucocorticoids); tissue hypoxia is likely at least partially responsible for this phenomena. In the present study, we found that acute exposure (24 h) of engineered muscle to either 1% or 5% O_2_ resulted in severe decrements in maximal contractile force generation. Similarly, myotube size was reduced under these O_2_ conditions compared to myotubes cultured under ambient O_2_ levels, and the loss of muscle size is also common in COPD sufferers [Mador and Bozkanat, 2001]. In the present study, we cannot discern whether hypoxia directly causes muscle wasting and impairments in muscle function, or whether this effect is indirectly mediated through the impact of hypoxia on other cellular processes (e.g., calcium handling, oxidative stress etc.); however, this response does closely match the phenotype observed in skeletal muscle of individuals with COPD, and particularly that observed during acute exacerbations [Gayan‐Ramirez and Decramer, 2013].

Elevations in the mRNA levels of the E3 ubiquitin ligases MuRF‐1 and MAFbx are common in a number of pathologies associated with muscle wasting [Gomes et al., 2001; Lecker et al., 2004] as well as muscle unloading [Bodine et al., 2001]. Furthermore, MuRF‐1 and MAFbx are elevated in skeletal muscle of individuals suffering from COPD compared to healthy individuals [Lemire et al., 2012] as well as in rodents exposed to hypoxia for 21 days [de Theije et al., 2013]. Interestingly, we found that MuRF‐1 mRNA was only elevated when engineered muscles were cultured at 1% O_2_, and no differences were seen above this level. By contrast, MAFbx mRNA expression was significantly increased when O_2_ levels were reduced to 10% or lower (10%, 5%, and 1%) suggesting that MAFbx is more sensitive to hypoxia than MuRF‐1. Since MuRF‐1/MAFbx expression can regulate muscle breakdown [Bodine et al., 2001], it is reasonable to suggest that the elevations in these ligases are at least partially responsible for the myotube atrophy and decrement in muscle function. Moreover, these data confirm that exposure of engineered muscle to hypoxia for 24 h activates a molecular pathway known to induce atrophy in skeletal muscle.

We also saw that p70S6 kinase phosphorylation (a downstream effector of mTORC1) was significantly blunted at 1% O_2_, suggesting that the loss of size and strength may be a consequence of decreased anabolic signaling as well as increased muscle catabolism. The role of p70S6 kinase in driving cellular hypertrophy is well defined [Shah et al., 2000], and it has also recently been shown that this protein kinase is required for increases in muscle force during hypertrophy [Marabita et al., 2016]. Furthermore, blunted signaling through the Akt‐mTORC1 axis has been shown in hypoxia treated cells [Caron et al., 2009], rodents [Favier et al., 2010; de Theije et al., 2013], and individuals suffering from COPD [Favier et al., 2010] as well as myotubes in vitro derived from COPD muscle [Pomiès et al., 2015]. This therefore suggests that the dampening of p70S6 kinase phosphorylation as a consequence of hypoxic exposure may in part instigate the loss of myotube size and muscle force production, and provides more evidence that engineered muscle exposed to hypoxia in vitro responds in a manner akin to that of diseased muscle and is comparable to other cell and animal models.

Since native muscle oxygenation lies at approximately 5% O_2_ [Richardson et al., 2006], 1% represents the only O_2_ level tested which would correspond to pathological tissue hypoxia in vivo. However, as myoblasts are routinely cultured at 21% O_2_ in vitro, any O_2_ level below this may be sensed by the cells as hypoxia and potentially cause alterations in physiological responses [Deldicque and Francaux, 2013]. It is therefore interesting that only small alterations were observed at 15% or 10% O_2_ at both the molecular and phenotypic level, and suggests that 5% O_2_ may represent a threshold at which muscle cells in vitro alter their homeostasis by reducing muscle size and thus strength. However, it is noteworthy that the increase in MuRF‐1 mRNA expression and reduction in p70S6 kinase phosphorylation were only apparent at 1% O_2_, and therefore pathological levels of hypoxia (1%) may exacerbate the molecular pathways driving skeletal muscle atrophy.

In summary, the present data suggest that engineered skeletal muscle exposed to hypoxia responds at both a phenotypic and molecular level in a manner akin to that observed in skeletal muscle of COPD patients. These findings go some way toward validating the physiological accuracy of such model systems, and suggest that engineered muscle may have utility in understanding the underlying pathophysiology of hypoxia associated muscle dysfunction. Furthermore, we have shown that the atrophy, loss of muscle function, and molecular responses to hypoxia are most pronounced when engineered muscle is cultured in O_2_ levels of 5% (physiological hypoxia) or less (pathological hypoxia), and thus an in vitro hypoxic threshold clearly exists for activation of a catabolic cellular response.
